# Supplementary material for: Innovation in Non-Invasive Diagnosis and Disease Monitoring for Meningiomas
Source: Int J Mol Sci. 2024 Apr 10;25(8):4195. doi: 10.3390/ijms25084195 (PMC11050588; doi:10.3390/ijms25084195)
Supplement: Supplementary file 1 [file ijms-25-04195-s001.zip › ijms-2901920-supplementary.pdf]

Table S1. Article search terms.

PubMed Searches:	Number of Results:	Google Scholar Searches:	Number of Results:
((meningioma) OR (brain tumor)) AND (((liquid biopsy) OR (genetic marker)) OR (biomarker)) AND (((cfDNA) OR (circulating tumor DNA)) OR (cell-free nucleic acids))	362	"meningioma" AND "liquid biopsy" AND "cfDNA"	188
((meningioma) OR (brain tumor)) AND ((liquid biopsy) OR (biomarker)) AND (extracellular vesicle)	341	"meningioma" AND "liquid biopsy" AND "EV"	169
(meningioma) AND (((liquid biopsy) OR (genetic markers)) OR (biomarker)) AND ((miRNA) OR (microRNAs))	32	"meningioma" AND "liquid biopsy" AND "miRNA"	255
(meningioma) AND (((seroreactivity) OR (serologic test)) OR (autoantibodies))	85	"meningioma" AND "liquid biopsy" AND ("seroreactivity" OR "autoantibody" OR "antigen")	328
((meningioma) OR (brain tumor)) AND (spectroscopy) AND ((liquid biopsy) OR (serum diagnostic))	124	"meningioma" AND "liquid biopsy" AND "spectroscopy"	156
